# Supplementary figures and images for: STAG1 vulnerabilities for exploiting cohesin synthetic lethality in STAG2-deficient cancers
Source: Life Sci Alliance. 2020 May 28;3(7):e202000725. doi: 10.26508/lsa.202000725 (PMC7266993; doi:10.26508/lsa.202000725)

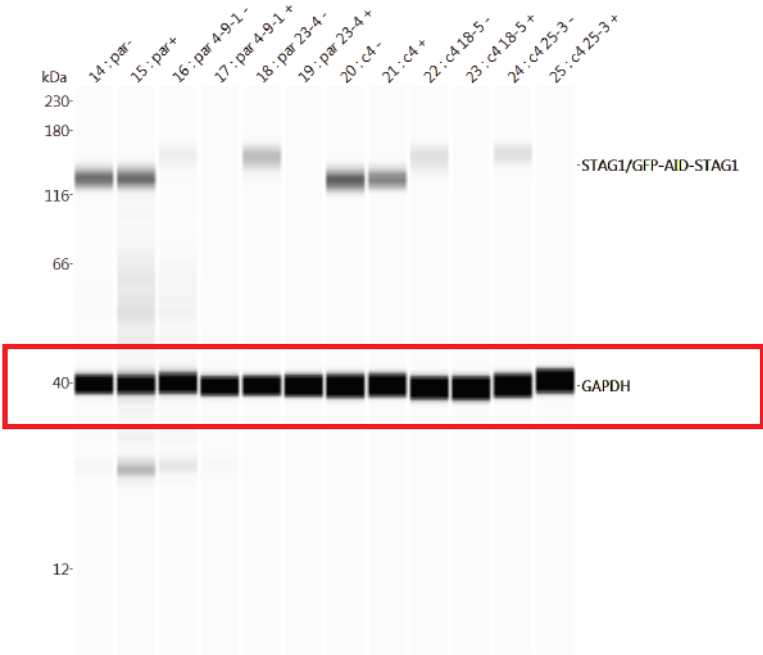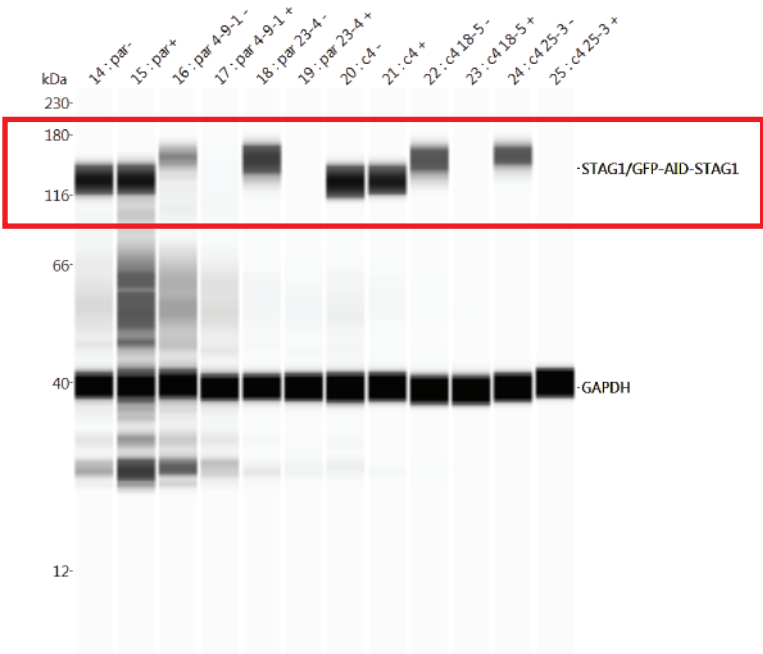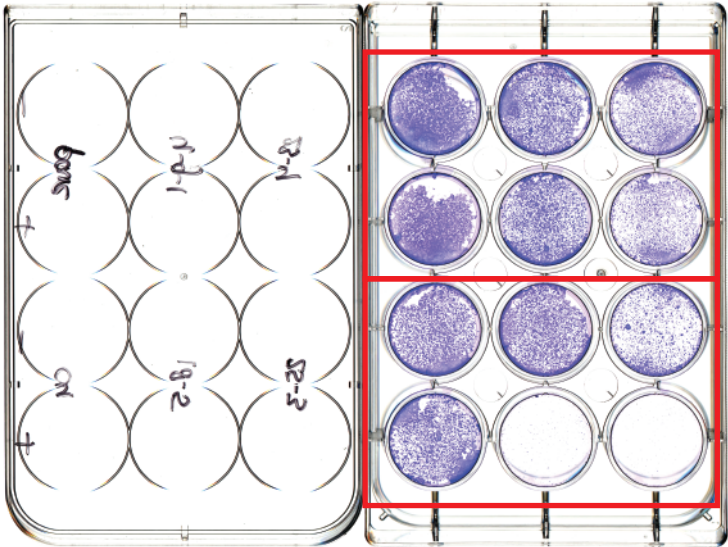

Supplement: Supplementary file 3 [file LSA-2020-00725_SdataF2.pdf]

B

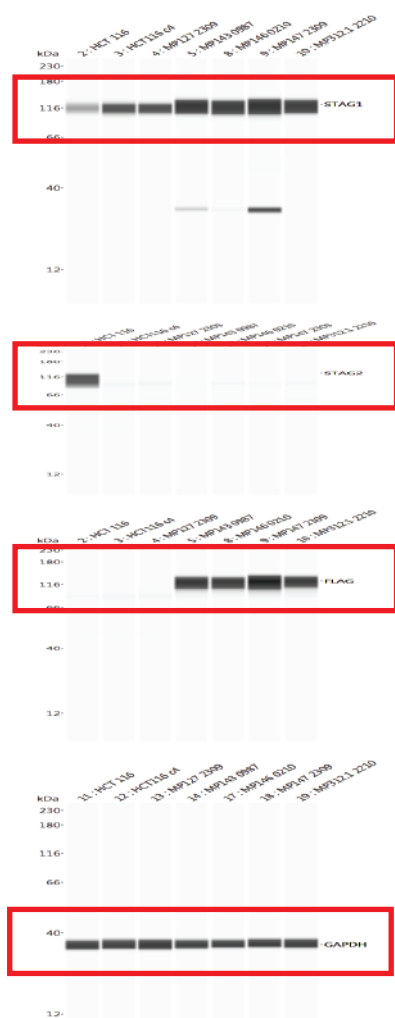

C

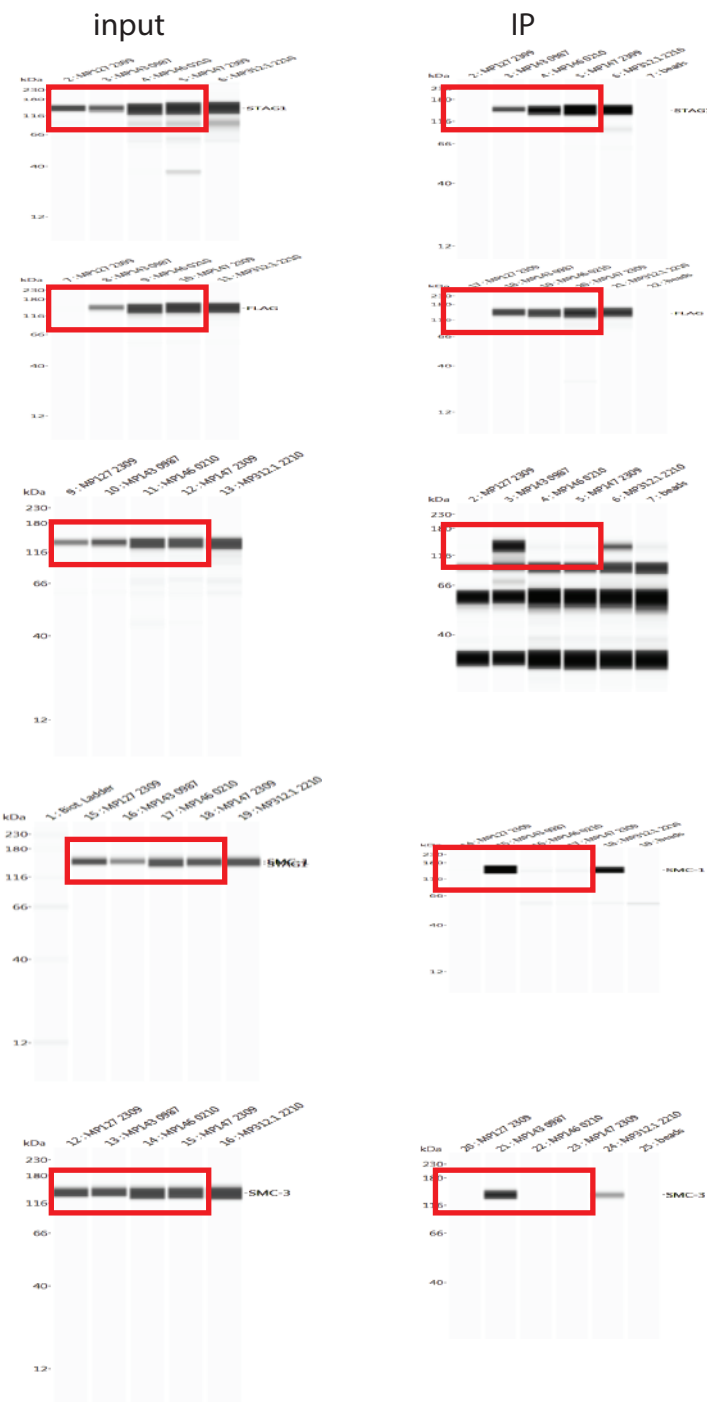

D

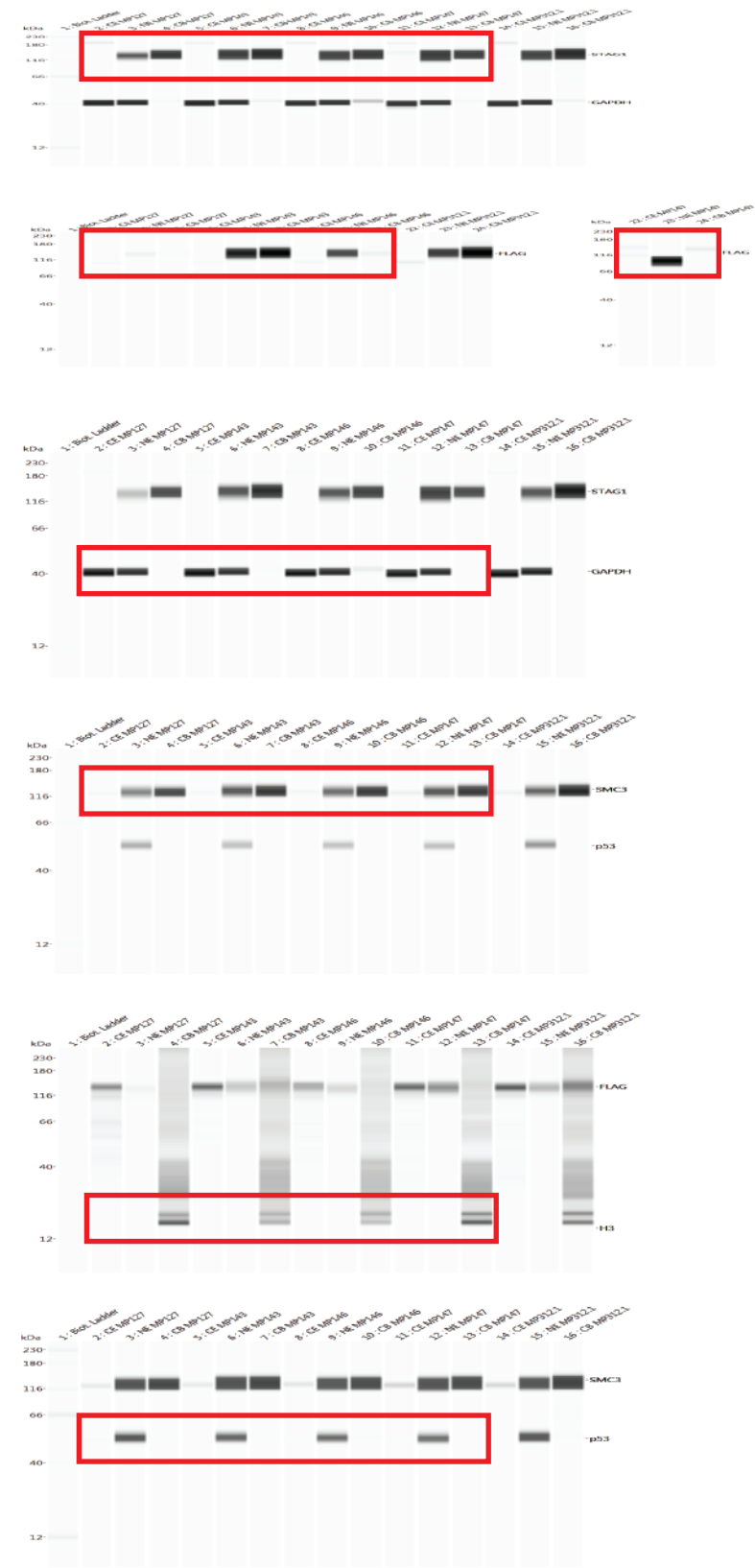

Supplement: Supplementary file 6 [file LSA-2020-00725_SdataF4.pdf]
