# Supplementary material for: STAG1 vulnerabilities for exploiting cohesin synthetic lethality in STAG2-deficient cancers
Source: Life Sci Alliance. 2020 May 28;3(7):e202000725. doi: 10.26508/lsa.202000725 (PMC7266993; doi:10.26508/lsa.202000725)
Supplement: Supplementary file 7 [file LSA-2020-00725_TableS3.docx]

Supplementary Table S3

**Cell lines used in this study**

| Cell line | Cancer | STAG2 status | Reference for STAG2 status | Source | STR confirmed |
| --- | --- | --- | --- | --- | --- |
| HCT 116 parental | Colon | Wild-type | (Solomon et al., 2011, van der Lelij et al., 2017) | ATCC | Yes |
| HCT 116 STAG2- 505c1 | Colon | M255fs (CRISPR KO) | (van der Lelij et al., 2017) | (van der Lelij et al., 2017) | Yes |
| HCT 116 STAG2- 502c4 | Colon | T220fs (CRISPR KO) | (van der Lelij et al., 2017) | (van der Lelij et al., 2017) | Yes |
| HCT 116 parental + GFP-AID-STAG1 cl1 | Colon | Wild-type | This study | This study | Yes |
| HCT 116 parental + GFP-AID-STAG1 cl4 | Colon | Wild-type | This study | This study | Yes |
| HCT 116 STAG2- 502c4 + GFP-AID-STAG1 cl3 | Colon | T220fs (CRISPR KO) | This study | This study | Yes |
| HCT 116 STAG2- 502c4 + GFP-AID-STAG1 cl5 | Colon | T220fs (CRISPR KO) | This study | This study | Yes |
| HCT 116 STAG2- 502c4 + FLAG-STAG1 wt | Colon | T220fs (CRISPR KO) | This study | This study | Yes |
| HCT 116 STAG2- 502c4 + FLAG-STAG1 D797K | Colon | T220fs (CRISPR KO) | This study | This study | Yes |
| HCT 116 STAG2- 502c4 + FLAG-STAG1 D797A | Colon | T220fs (CRISPR KO) | This study | This study | Yes |
| KBM-7 B4 | Leukemia | Wild-type | This study | This study | Yes |
| KBM-7 *STAG2*- c9 | Leukemia | T220fs (CRISPR KO) | This study | This study | Yes |
| KBM-7 STAG2- c11 | Leukemia | M163fs (CRISPR KO) | This study | This study | Yes |
| UM-UC-3 | Bladder | K983* | (Solomon, Kim et al., 2013) | ATCC | Yes |
| RT-112 | Bladder | Wild-type | (Benedetti et al., 2017) | CLS cell line service | Yes |
